# Tetraploid Citrumelo 4475 rootstocks improve diploid common clementine tolerance to long-term nutrient deficiency

**DOI:** 10.1038/s41598-021-88383-5

**Published:** 2021-04-26

**Authors:** Julie Oustric, Stéphane Herbette, Yann Quilichini, Raphaël Morillon, Jean Giannettini, Liliane Berti, Jérémie Santini

**Affiliations:** 1 CNRS, Équipe de Biochimie et Biologie Moléculaire du Végétal, UMR 6134 SPE, Université de Corse, Corsica, France; 2 UCA, INRAE, PIAF, Clermont-Ferrand, France; 3 CNRS, Équipe des Parasites et Ecosystèmes Méditerranéens, UMR 6134 SPE, Université de Corse, Corsica, France; 4 Equipe SEAPAG, CIRAD, UMR AGAP, Petit-Bourg, 97170 Guadeloupe, France; 5 AGAP, CIRAD, INRAE, Institut Agro, Univ Montpellier, Montpellier, France

**Keywords:** Biochemistry, Physiology, Plant sciences, Environmental sciences

## Abstract

Nutrient deficiency alters growth and the production of high-quality nutritious food. In Citrus crops, rootstock technologies have become a key tool for enhancing tolerance to abiotic stress. The use of doubled diploid rootstocks can improve adaptation to lower nutrient inputs. This study investigated leaf structure and ultrastructure and physiological and biochemical parameters of diploid common clementine scions (C) grafted on diploid (2x) and doubled diploid (4x) Carrizo citrange (C/CC2x and C/CC4x) and Citrumelo 4475 (C/CM2x and C/CM4x) rootstocks under optimal fertigation and after 7 months of nutrient deficiency. Rootstock ploidy level had no impact on structure but induced changes in the number and/or size of cells and some cell components of 2x common clementine leaves under optimal nutrition. Rootstock ploidy level did not modify gas exchanges in Carrizo citrange but induced a reduction in the leaf net photosynthetic rate in Citrumelo 4475. By assessing foliar damage, changes in photosynthetic processes and malondialdehyde accumulation, we found that C/CM4x were less affected by nutrient deficiency than the other scion/rootstock combinations. Their greater tolerance to nutrient deficiency was probably due to the better performance of the enzyme-based antioxidant system. Nutrient deficiency had similar impacts on C/CC2x and C/CC4x. Tolerance to nutrient deficiency can therefore be improved by rootstock polyploidy but remains dependent on the rootstock genotype.

## Introduction

Fruit crops, especially citrus fruits, require large amounts of fertilizers to ensure good production and fruit quality. Today, the challenge for sustainable agriculture, and particularly organic agriculture, is to reduce the use of inputs in crops. Reducing inputs optimizes the economic outcome while limiting the environmental impact.

Water and minerals absorbed by roots in the soil are essential for plant development, growth and reproduction. Fourteen minerals are considered essential and these can be divided into two groups: the macroelements (N, K, P, Ca, Mg and S) which are constituents of organic matter (proteins, nucleic acids) or play a strong osmotic role, and microelements (Zn, Cu, Fe, Mn, B, Mo, Cl and Ni), which are only involved as specific cofactors or constituents of certain enzymes^1–3^. Mineral-deficient plants present various visual symptoms, such as necrosis, chlorosis, dark green foliage, or stunted growth^4^. Essential mineral deficiency alters plant primary metabolism and this disrupts the physiological and biochemical processes leading to changes in leaf structure and ultrastructure^5,6^. Moreover, cells must cope with an overproduction of reactive oxygen species (ROS) such as singlet oxygen (O^*^), hydroxyl radicals (OH^·^), superoxide anion (O_2_^·−^) and hydrogen peroxide (H_2_O_2_) which cause membrane leakage due to lipid peroxidation and damage to proteins and nucleic acids^7–9^. As a result, ROS defence mechanisms are activated by a set of antioxidant compounds (metabolites such as ascorbate and proline) and antioxidant enzymes (superoxide dismutase (SOD), catalase (CAT), ascorbate peroxidase (APX) and dehydroascorbate reductase (DHAR)). Adapted genotypes are therefore sought in order to propose cultural itineraries more suitable to low input conditions.

In citrus crops, improving the performance of varieties is based on the scion/rootstock combination forming the aerial parts and roots of the plant, respectively. The impact of the rootstock on the scion lies in its influence on the plant growth and development and the adaptation to environmental conditions^10^. Grafting improves the agronomic fruit quality by increasing synthesis of endogenous hormones and the acquisition and transport of mineral nutrient^11,12^. It also influences the organoleptic quality by impairing secondary metabolites which impacts the flavour^11,13^ and the content of fruit metabolites^14,15^. An efficient translocation of water and mineral nutrients between rootstock and scion promote biomass production and tolerance to biotic and abiotic factors, such as nutrient deficiency^16,17^. Improved root system vigour in citrus rootstocks results in increased soil nutrient and water uptake^15,18^. All modern cultivated varieties of citrus are now grafted on diploid rootstocks and therefore have two sets of chromosomes in their genetic heritage. However, incomplete mitosis of somatic embryos may occur in seedlings of diploid (2x) apomictic genotypes with formation of doubled diploid (4x) genotypes^19^.

Tetraploidy modifies phenotypic characteristics such as size and density of stomata, root and leaf morphology as well as growth, development and quality of fruits^20^. These changes may result in an upheaval in physiological processes^21^. Studies have shown that tetraploid citrus rootstocks can lead to a reduction in yield without changing the quality of the fruit^22–24^. Recent advances have shown an interesting impact of the rootstock tetraploidy on the scion tolerance to environmental stresses. For example, grafting of Valencia Delta sweet orange (*Citrus sinensis* L.) on 4x rangpur lime (*Citrus limonia*) rootstock was found to improve its tolerance to water stress by changing patterns of gene expression in Rangpur lime citrus roots regulating adaptation to water deficit^25^. Natural chilling stress tolerance associated with a robust antioxidant system was also enhanced in 2x common clementine (*Citrus clementina* Hort. ex Tan) grafted with Carrizo citrange (*Citrus sinensis* Osb. × *Poncirus trifoliata* L. Raf.) 4x rootstock^26^. Chromium tolerance of Kinnow mandarin (*Citrus nobilis* Lour x *Citrus deliciosa* Ten) grafted on three 4x rootstocks *(Poncirus trifoliata* [L.], *Citrus reshni,* and *Citrus limonia* Osbeck.) may be attributed to chromium sequestration in roots with lower transfer to leaves in 4x rootstocks^27^. The use of rootstocks better adapted to environmental constraints seems to be a promising eco-friendly strategy.

Many *Citrus* genotypes are used as rootstock for citrus cultivation. Genotypes belong either to the *Citrus* genus such as Volkamer lemon or are obtained by hybridization between *Citrus* and *Poncirus* genus progenitors such as Citrumelo 4475 and Carrizo citrange. Volkamer lemon which is used as rootstock for lemon, is adapted to dry, calcareous and saline soils and presents tolerance to Tristeza, cachexia and exocortis. Citrumelo 4475 imparts cold tolerance to the scion. Carrizo citrange is frequently used in acidic and neutral soils but not in dry areas because of its limited performance under drought conditions. These two genotypes inherited Tristeza tolerance from their Trifoliate orange progenitor and give clementine varieties that produce a high yield and fruit quality^28^.

In a previous study, we compared leaf structure and ultrastructure under nutrient deficiency of two genotypes used worldwide as rootstock for citrus cultivation, the Citrumelo 4475 (*Citrus paradisi* L. Macf. × *Poncirus trifoliata* L. Raf.) and Volkamer lemon (*Citrus limonia* Osb.) with both 2x and 4x genotypes^29^. Results showed an increase in tolerance to nutrient deficiency in 4x genotypes. Doubled diploid genotypes presented less degradation of ultrastructural components such as chloroplasts, thylakoids, mitochondria and starch grains associated with a smaller decrease in leaf net photosynthetic rate (*P*_net_), stomatal conductance (*g*_s_) and chlorophyll fluorescence (*F*_v_/*F*_m_) compared to 2x genotypes.

The higher tolerance to nutrient deficiency was linked to the reduced accumulation of malondialdehyde (MDA) and H_2_O_2_ in Citrumelo 4475 4x than in the 2x, implying a more efficient antioxidant system in the 4x genotype. However, few differences in the antioxidant system and oxidative status were observed between 2x and 4x Volkamer lemons.

The aim of this study was therefore to determine the impact of three combined factors, (i) the rootstock genotypes (ii), the rootstock ploidy level and (iii) the nutrient deficiency on the 2x common clementine scion. We selected 2x common clementine grafted on two rootstocks used worldwide for clementine cultivation, i.e. Citrumelo 4475 (C/CM2x) and Carrizo citrange (C/CC2x) in both 2x and 4x types (C/CM4x and C/CC4x, respectively). The effect of rootstock ploidy level on 2x common clementine scions under nutrient deficiency was investigated by studying 2x common clementine leaf structure and ultrastructure and physiological and biochemical parameters.

## Results and discussion

Effect of rootstock ploidy level on anatomical properties of 2x common clementines under control conditions.

**Figure 1:**
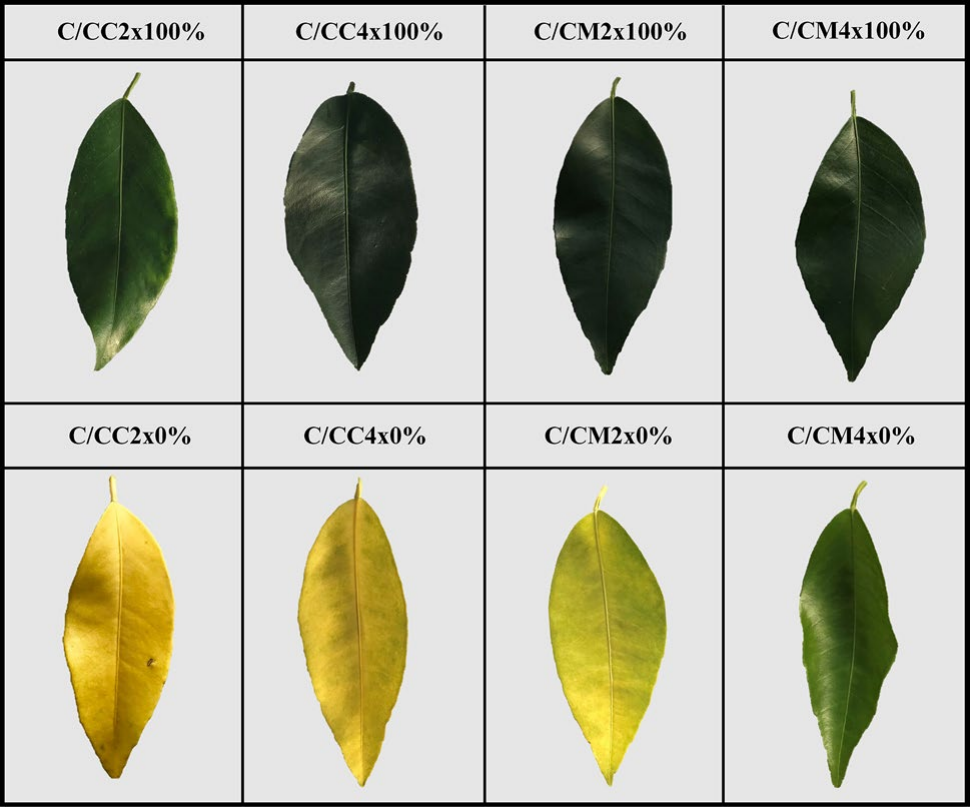
Leaf damages of common clementine scion grafted onto different rootstocks. Leaf damages of common clementine scion grafted onto diploid (C/CC2x) and doubled diploid (C/CC4x) Carrizo citrange and diploid (C/CM2x) and doubled diploid (C/CM4x) Citrumelo 4475 rootstocks grown in nutrient reference solution (100%) and without nutrient solution (0%).

**Figure 2:**
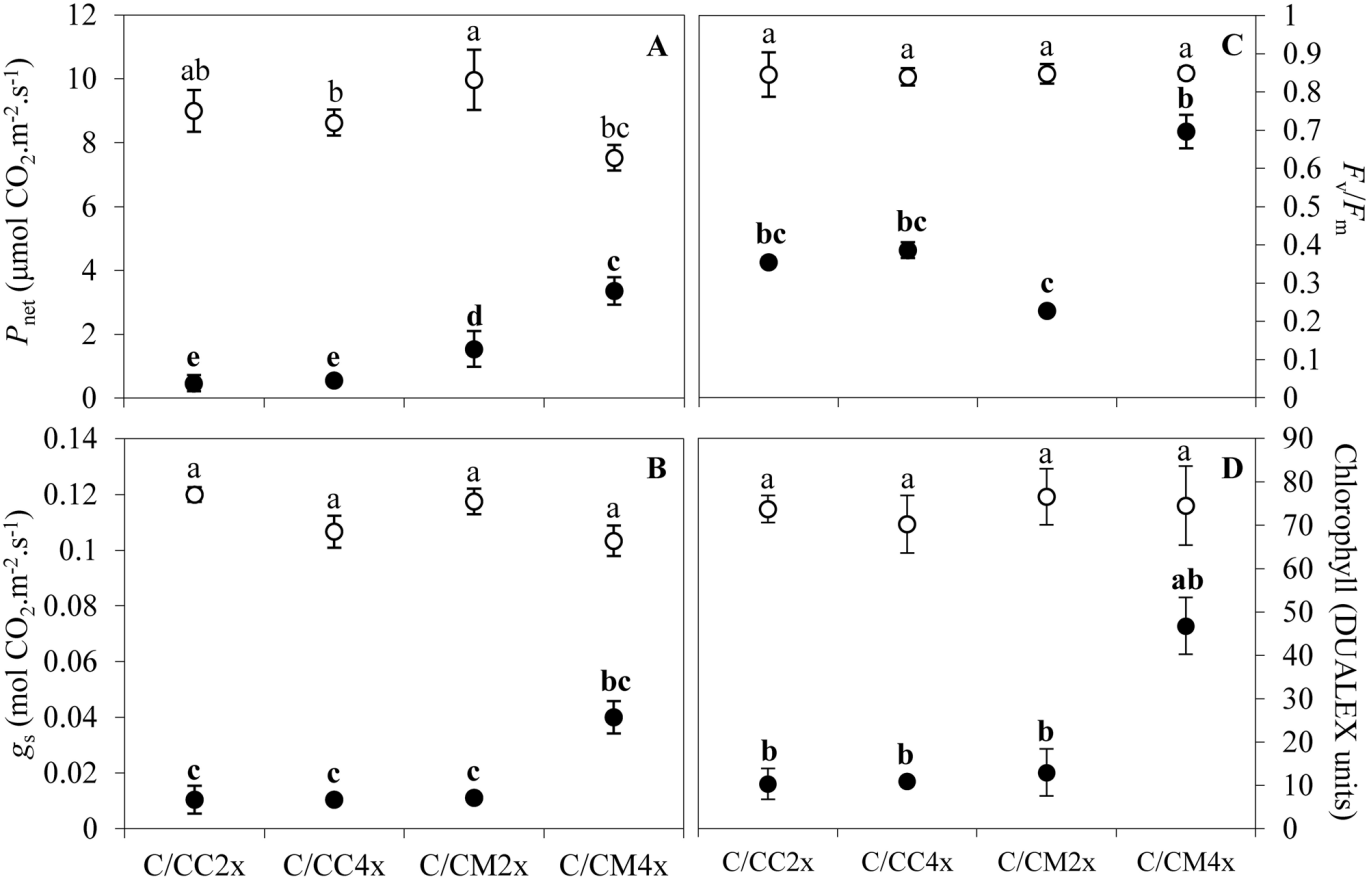
Effect of nutrient deficiency on photosynthetic properties of leaves of common clementine scion grafted onto different rootstocks. (**A**) Leaf net photosynthetic rate (*P*_net_), (**B**) stomatal conductance (*g*_s_), (**C**) chlorophyll fluorescence (*F*_v_/*F*_m_) and (**D**) chlorophyll contents of leaves of common clementine scion grafted onto diploid (C/CC2x) and doubled diploid (C/CC4x) Carrizo citrange and diploid (C/CM2x) and doubled diploid (C/CM4x) Citrumelo 4475 rootstocks grown in nutrient reference solution (100%) (white circles) and without nutrient solution (0%) (black circles) for 210 days. Values are mean (± standard error) of 9 independent measurements (*n* = 9) for each scion/rootstock combination, i.e. three per tree. Ploidy and treatment effects were analyzed using ANOVA and Fisher LSD tests (*p* < 0.05). Distinct letters indicate significant differences between all scion/rootstocks combinations and treatments.

All the scion/rootstock combinations present similar leaves colour, N contents and chlorophyll contents (Figs. 1, 2D, Table 1).

**Table 1:** Total content of macronutrient in leaves of common clementine scion grafted onto different rootstocks.

Nutrients	C/CC2x		C/CC4x		C/CM2x		C/CM4x	
	100%	0%	100%	0%	100%	0%	100%	0%
N (%)	2.98 ± 0.15^a^	1.12 ± 0.06^b^	3.39 ± 0.44^a^	1.19 ± 0.05^b^	3.32 ± 0.04^a^	1.15 ± 0.05^b^	3.90 ± 0.57^a^	1.28 ± 0.03^b^
P (%)	0.15 ± 0.01^b^	0.43 ± 0.02^a^	0.18 ± 0.01^b^	0.38 ± 0.02^a^	0.15 ± 0.00^b^	0.16 ± 0.01^b^	0.14 ± 0.00^b^	0.20 ± 0.01^b^
K (%)	2.60 ± 0.15^b^	4.85 ± 0.10^a^	2.21 ± 0.00^b^	4.13 ± 0.04^a^	1.79 ± 0.08^c^	2.79 ± 0.26^b^	2.34 ± 0.22^b^	2.33 ± 0.10^b^
Ca (%)	1.61 ± 0.19^ab^	1.85 ± 0.15^a^	1.32 ± 0.15^c^	1.42 ± 0.08^bc^	1.67 ± 0.30^ab^	1.63 ± 0.11^ab^	1.65 ± 0.21^ab^	1.58 ± 0.10^b^
Mg (%)	0.61 ± 0.02^ cd^	0.89 ± 0.04^ab^	0.69 ± 0.07^c^	0.80 ± 0.01^b^	0.54 ± 0.03^d^	0.68 ± 0.06^c^	0.87 ± 0.02^ab^	0.96 ± 0.08^a^
Na (%)	0.016 ± 0.001^b^	0.040 ± 0.002^ab^	0.014 ± 0.000^b^	0.063 ± 0.004^a^	0.014 ± 0.001^b^	0.019 ± 0.000^b^	0.018 ± 0.001^b^	0.018 ± 0.000^b^

Nitrogen (N), phosphorus (P), potassium (K), magnesium (Mg), calcium (Ca) and sodium (Na) content in leaves of common clementine scion grafted onto diploid (C/CC2x) and doubled diploid (C/CC4x) Carrizo citrange and diploid (C/CM2x) and doubled diploid (C/CM4x) Citrumelo 4475 rootstocks grown in nutrient reference solution (100%) and without nutrient solution (0%). Values are means (± standard error) of 3 independent measurements from 3 samples for each scion/rootstock, i.e. one per tree (*n* = 3). One sample was obtained by pooling 8 fully-expanded leaves. Data were analyzed using ANOVA and Fisher LSD tests (*p* < 0.05).

Different letters indicate significant differences between the four scion/rootstock combinations and treatments.

It is understandable as N is a structural element of chlorophyll and thereby affects leaf colour^30–32^. P and Na contents were also similar between all scion/rootstock combinations while K and Ca contents were lower in C/CM2x and C/CC4x compared to the other scion/rootstock combination. Considering the Mg content, it was different between the 2x common clementine grafted onto Citrumelo 4475 and Carrizo citrange and between ploidy level for Citrumelo 4475. These results suggest that the rootstock genotypes and their ploidy level can influence the nutrient minerals content in the scion leaves.

Whatever the rootstock genotype and ploidy level, microscopic examination of leaf surface imprints confirmed the presence of stomata only on their abaxial surface that were surrounded by ordinary epidermal cells^33^ (anomocytic organization) (Fig. 1). Rootstock tetraploidy did not induce any changes in the location of stomata or the epidermal cell structure of 2x common clementine leaves. This agrees with this anomocytic organization observed on leaves of tetraploid seedlings^29^.

**Figure 3:**
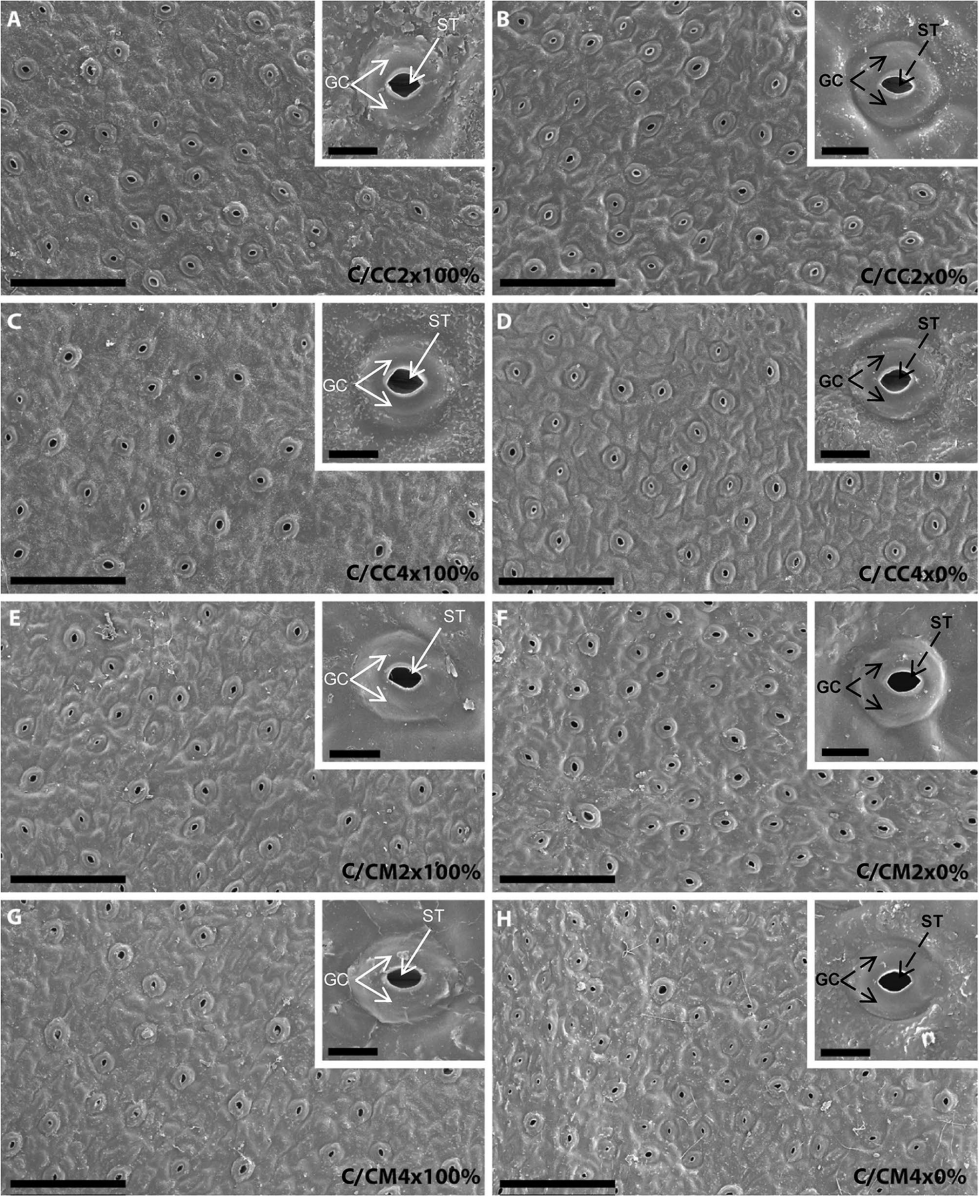
Scanning electron micrographs of abaxial epidermis and stomata in leaves of common clementine scion grafted onto different rootstocks. Abaxial epidermis and stomata of leaves of common clementine scion grafted onto diploid (C/CC2x) and doubled diploid (C/CC4x) Carrizo citrange and diploid (C/CM2x) and doubled diploid (C/CM4x) Citrumelo 4475 rootstocks grown in nutrient reference solution (100%) (**A,C,E,G**) and without nutrient solution (0%) (**B,D,F,H**) for 210 days. Scale bars (10 µm) are indicated at the bottom left for each image. The significant values between rootstock ploidy level of scion/rootstock combinations and treatment are shown in black and bold with a black dotted arrow. *ST* ostiole (stoma), *GC* guard cells.

Stomata size was unchanged in 2x common clementine scion regardless of the rootstocks ploidy level but was higher with Citrumelo 4475 than Carrizo citrange rootstock (Fig. 3, Table 2, Supplementary Table S1). Rootstock genotype influence the stomata density of the 2x common clementine scion but not the ostiole size. Stomata density and ostiole size was influenced by the rootstock ploidy level in Carrizo citrange with respectively a decrease and increase in C/CC4x compared to C/CC2x combinations (Fig. 3, Table 2, Supplementary Table S1).

**Table 2:**
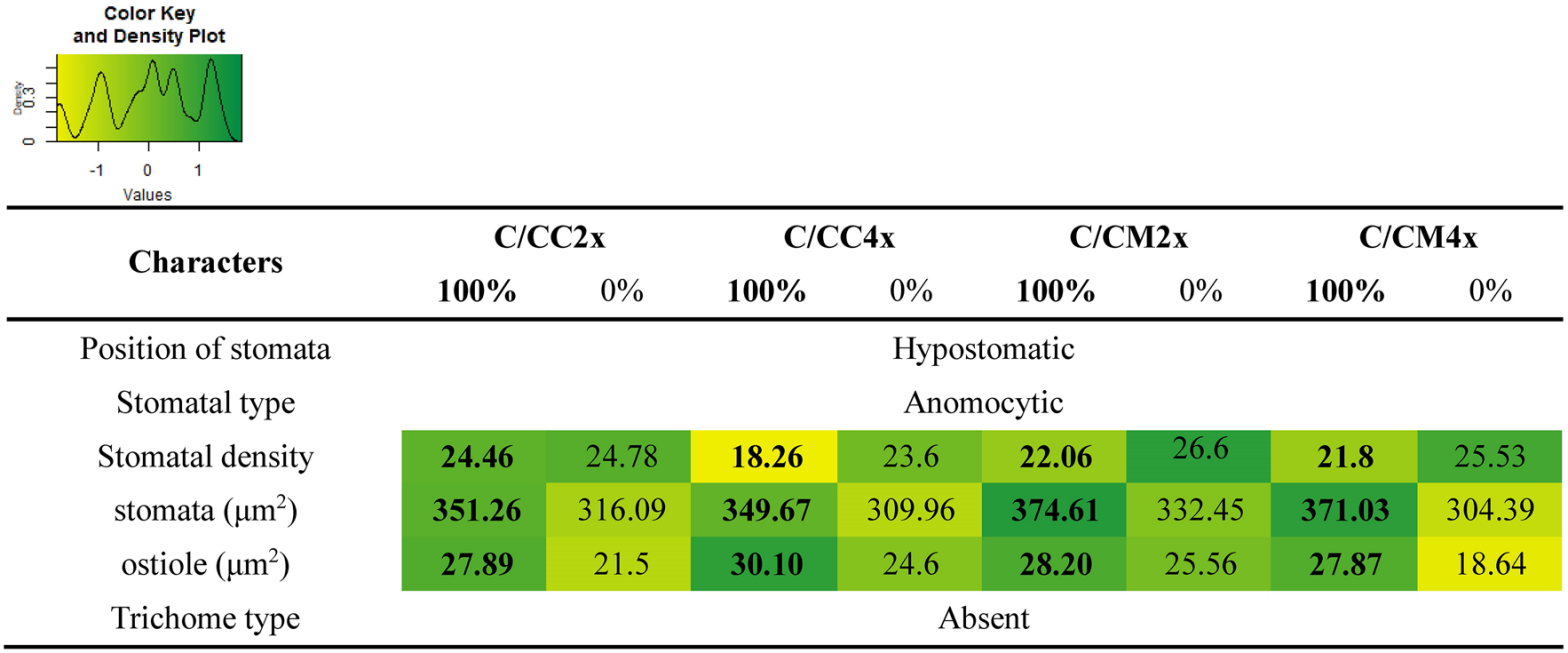
Anatomy of the leaf epidermis of common clementine scion grafted onto different rootstock.

Anatomical characters of leaves epidermis of common clementine scion grafted onto diploid (C/CC2x) and doubled diploid (C/CC4x) Carrizo citrange and diploid (C/CM2x) and doubled diploid (C/CM4x) Citrumelo 4475 rootstocks grown in nutrient reference solution (100%) and without nutrient solution (0%) for 210 days. For length and width of stomata and ostioles, values are means (± standard error) of 30 independent measurements on three different leaves (*n* = 90). For stomatal density, values are means of five independent measurements on three different leaves (*n* = 15) for stomatal density. The heatmap shows the differences between the four scion/rootstocks combinations and treatments for each characteristic. Values are associated with color ranging from yellow (low) to dark green (high).

Studies have shown a positive correlation between stomata and ostiole size and a negative correlation between stomata density and ploidy level in both 4x ungrafted genotypes and 3x clementine in comparison to their 2x counterparts^29,33,34^. Our study showed that 4x rootstocks had no effect on stomata sizes in 2x common clementine scions and a non-systematic impact on ostiole size and stomatal density, putatively through changes in hydraulics or mineral inputs. The decrease in stomatal density caused by rootstock tetraploidy in the C/CC4x combination was not associated with any changes in gas exchanges compared to its C/CC2x counterpart (Figs. 2, 3, Table 2, Supplementary Table S1). The increase in ostiole size in the C/CC4x combination compared to its C/CC2x counterpart suggests an adjustment of the stomata opening to compensate for the reduced stomatal density and maintain stomatal conductance^35^. This adjustment could be due to the considerable degradation of starch grains in the guard cells in the first hour of light contributing to a rapid increase in the opening of the stomata in parallel with the activation of membrane ion transport^36,37^.

Despite identical physical stomatal attributes between C/CM2x and C/CM4x combinations, analysis of the gas exchange parameters revealed a decrease of *P*_net_ in the C/CM4x combination (Figs. 2, 3, Table 2, Supplementary Table S1). Other factors than leaf structure therefore appear to be involved in the regulation of photosynthesis in the C/CM4x combination.

**Figure 4:**
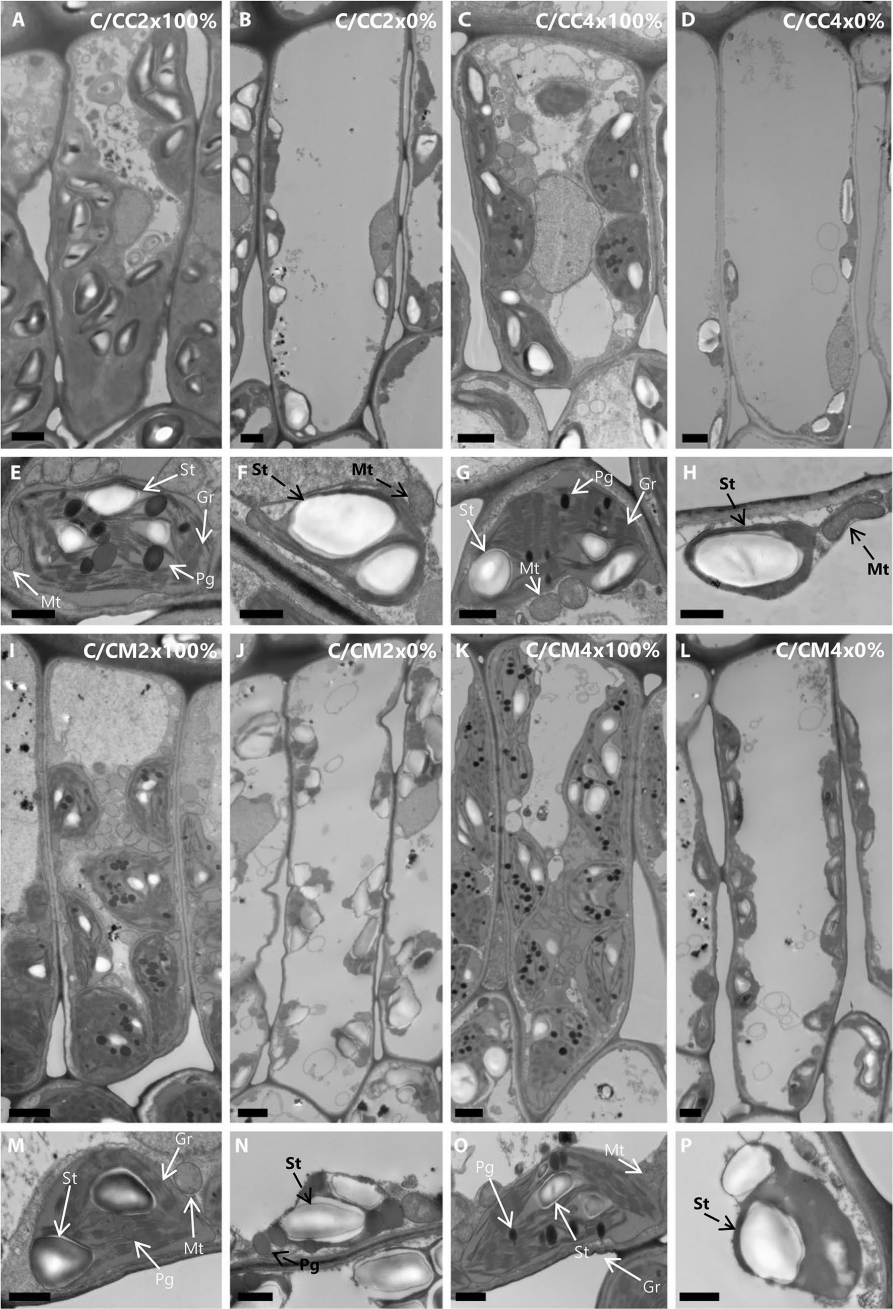
Transmission electron micrographs of palisade mesophyll cells in leaves of common clementine scion grafted onto different rootstocks. ((**A,B,C,D,I,J,K,L**); scale bar: 5 µm) Palisade mesophyll cells and ((**E,F,G,H,M,N,O,P**); scale bar: 1 µm) their respective chloroplasts in leaves of common clementine scion grafted onto diploid (C/CC2x) and doubled diploid (C/CC4x) Carrizo citrange and diploid (C/CM2x) and doubled diploid (C/CM4x) Citrumelo 4475 rootstocks grown in nutrient reference solution (100%). Scale bars for palisade mesophyll cells (5 µm) and their respective chloroplasts in leaves (1 µm) are indicated at the bottom left for each image. The significant values between rootstock ploidy level of scion/rootstock combinations and treatment are shown in bold and black with a black dotted arrow. *St* starch, *Pg* plastoglobuli, *Mt* mitochondria, *Gr* granum.

**Figure 5:**
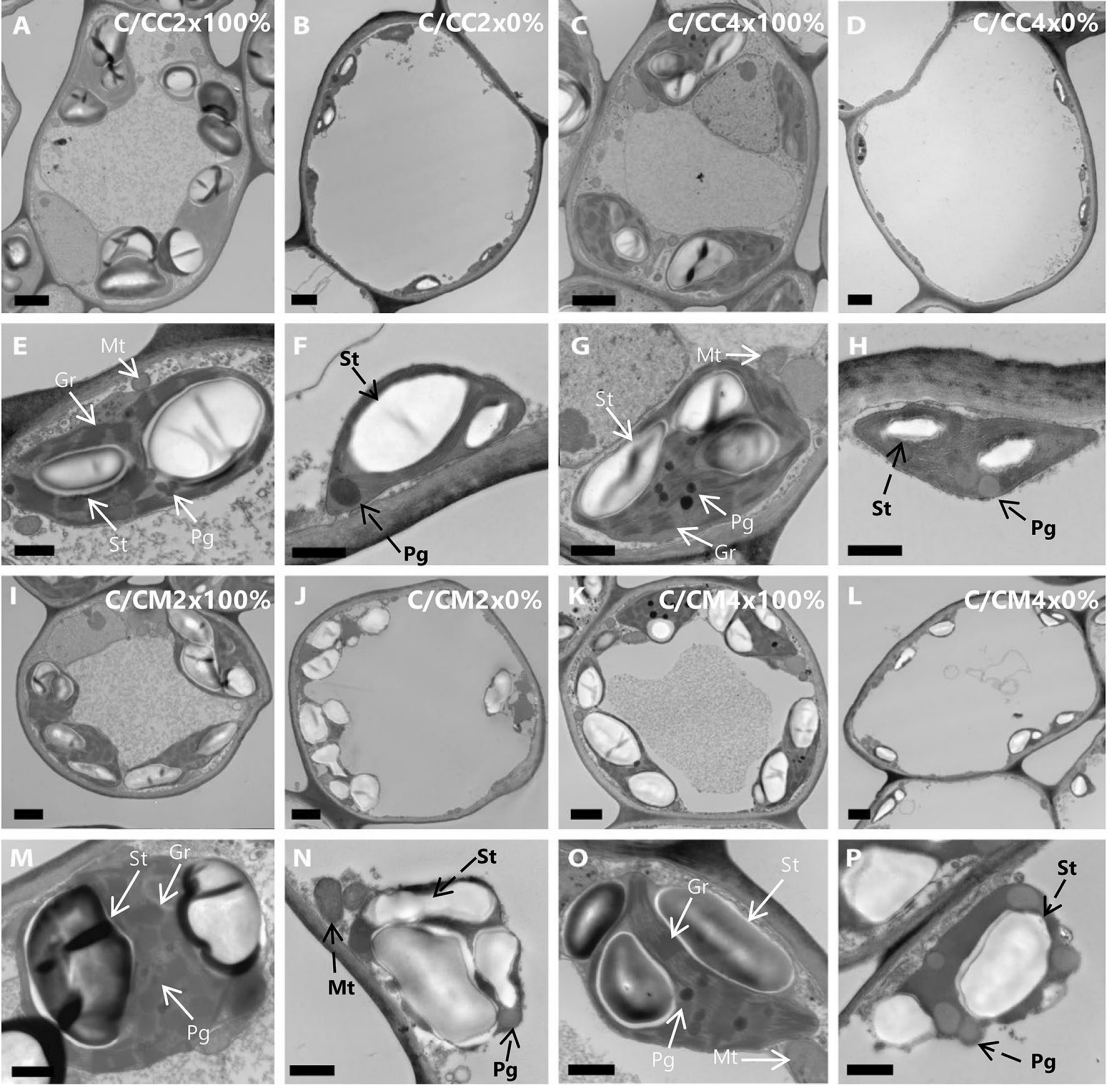
Transmission electron micrographs of spongy mesophyll cells of leaves of common clementine scion grafted onto different rootstocks. ((**A,B,C,D,I,J,K,L**); scale bar: 5 µm) Spongy mesophyll cells and ((**E,F,G,H,M,N,O,P**); scale bar: 1 µm) their respective chloroplasts in leaves of common clementine scion grafted onto diploid (C/CC2x) and doubled diploid (C/CC4x) Carrizo citrange and diploid (C/CM2x) and doubled diploid (C/CM4x) Citrumelo 4475 rootstocks grown in nutrient reference solution (100%). Scale bars for spongy mesophyll cells (5 µm) and their respective chloroplasts in leaves (1 µm) are indicated at the bottom left for each image. The significant values between rootstock ploidy level of scion/rootstock combinations and treatment are shown in bold and black with a black dotted arrow. *St* starch, *Pg* plastoglobuli, *Mt* mitochondria, *Gr* granum.

At ultrastructural level, genotypes and ploidy level influence the cell size and thickness in palisade and mesophylls as indicated by their reduction in C/CC4x compared to C/CC2x, and their increase in C/CM4x compared to C/CM2x (Figs. 4, 5, Tables 3, 4, Supplementary Tables S2, S3).

**Table 3:**
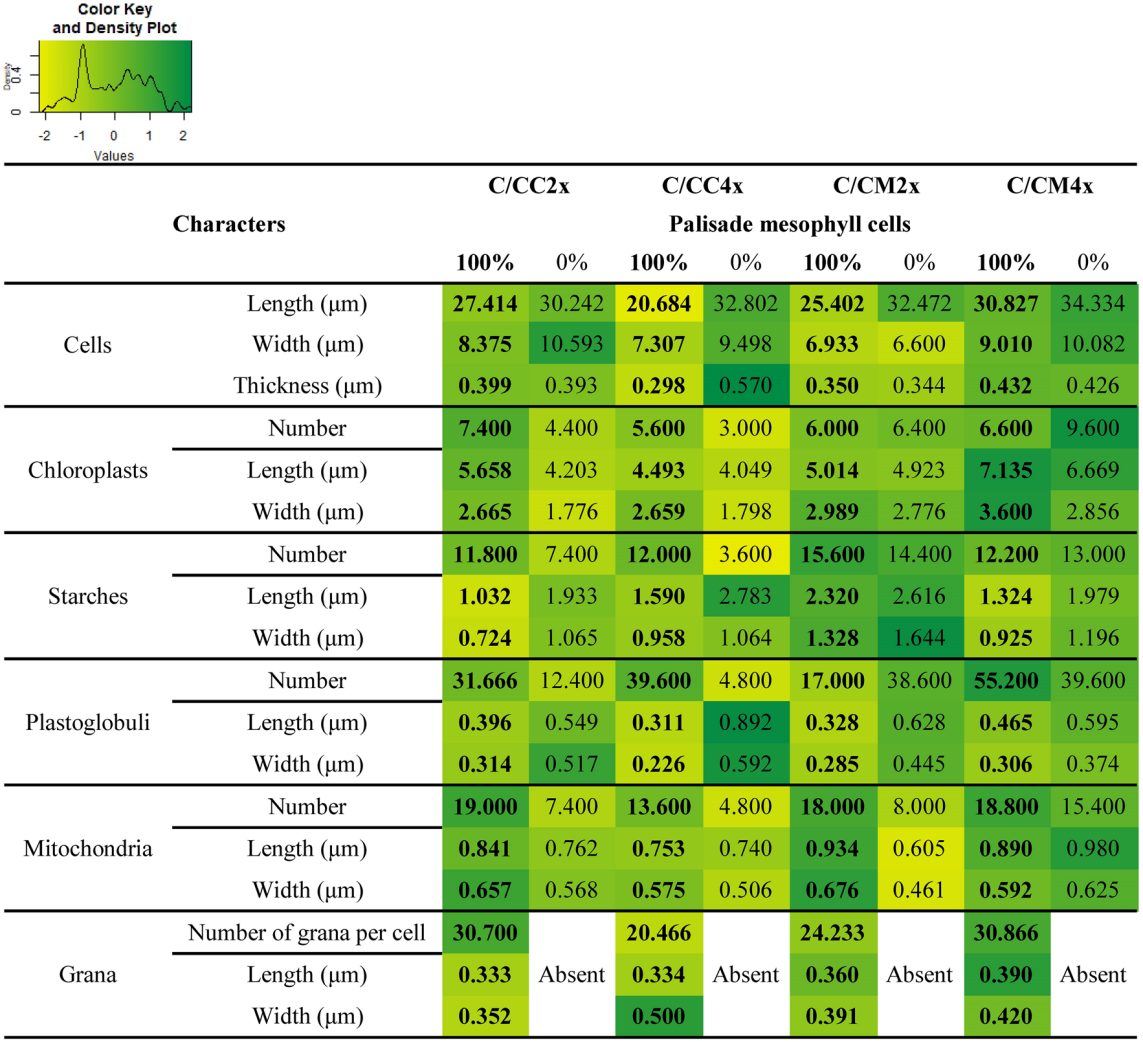
Ultrastructure of palisade mesophyll cells of leaves of common clementine scion grafted onto different rootstocks.

Ultrastructure characters of palisade mesophyll cells of leaves of common clementine scion grafted onto diploid (C/CC2x) and doubled diploid (C/CC4x) Carrizo citrange and diploid (C/CM2x) and doubled diploid (C/CM4x) Citrumelo 4475 rootstocks grown in nutrient reference solution (100%) and without nutrient solution (0%) for 210 days. Values are means (± standard error) of independent measurements on five different cells section (*n* = 5) for length, width and thickness of cells and for number of chloroplasts, starches, plastoglobuli and mitochondria and of 30 independent measurements on different cells section (*n* = 30) for length and width of chloroplasts, starches, plastoglobuli, mitochondria, grana, number of grana per cells section and number of thylakoids per granum. The heatmap shows the differences between the four scion/rootstocks combinations and treatments for each characteristic. Values are associated with color ranging from yellow (low) to dark green (high).

**Table 4:**
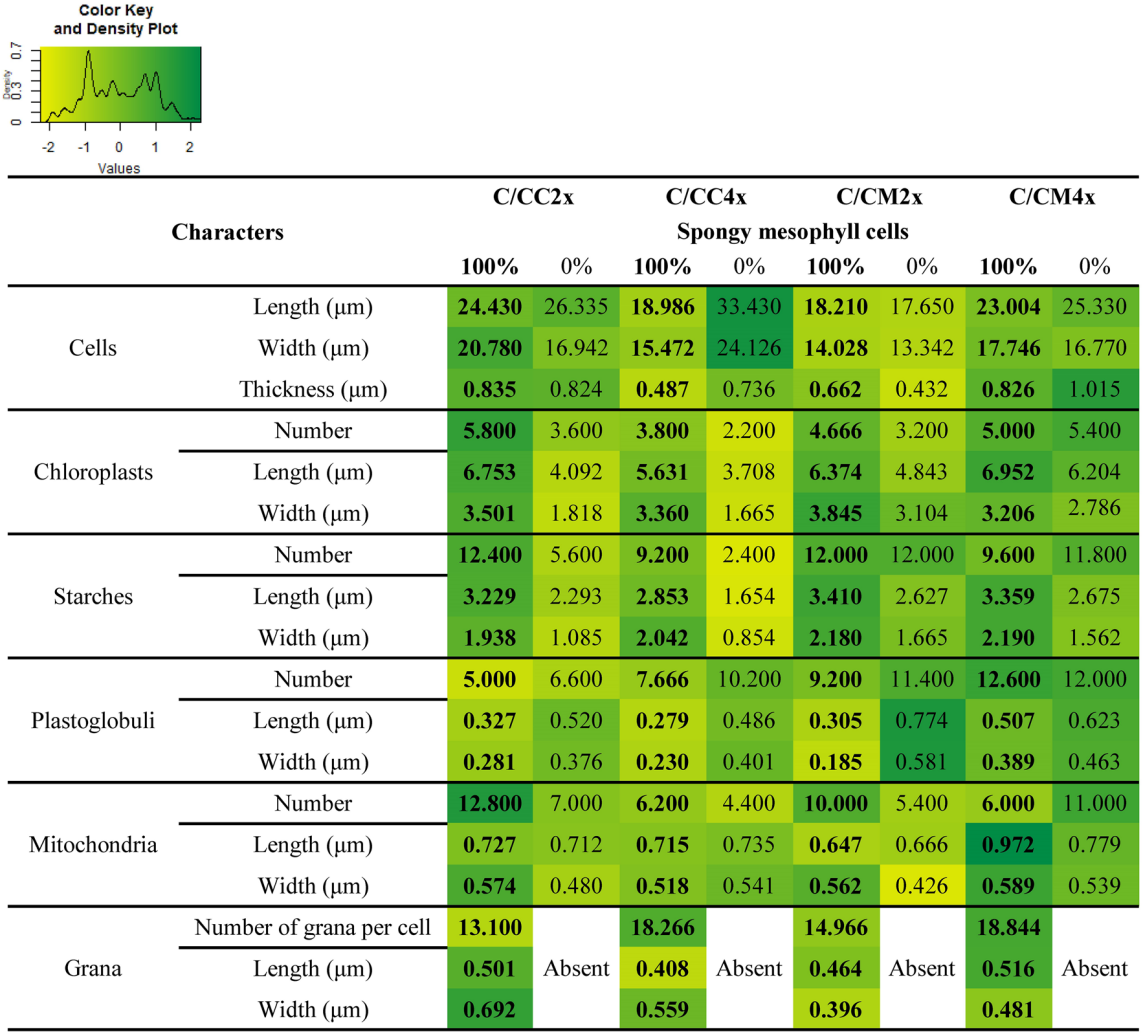
Ultrastructure of spongy mesophyll cells of leaves of common clementine scion grafted onto different rootstocks.

Ultrastructure characters of spongy mesophyll cells of leaves of common clementine scion grafted onto diploid (C/CC2x) and doubled diploid (C/CC4x) Carrizo citrange and diploid (C/CM2x) and doubled diploid (C/CM4x) Citrumelo 4475 rootstocks grown in nutrient reference solution (100%) and without nutrient solution (0%) for 210 days. Values are means (± standard error) of independent measurements on five different cells section (*n* = 5) for length, width and thickness of cells and for number of chloroplasts, starches, plastoglobuli and mitochondria and of 30 independent measurements on different cells section (*n* = 30) for length and width of chloroplasts, starches, plastoglobuli, mitochondria, grana, number of grana per cells section and number of thylakoids per granum. The heatmap shows the differences between the four scion/rootstocks combinations and treatments for each characteristic. Values are associated with color ranging from yellow (low) to dark green (high).

These ultrastructural modifications were the only identical changes in C/CM4x and the ungrafted rootstock counterpart^29^. In contrast to its ungrafted counterpart, C/CM4x showed a lower *P*_net_ and similar chloroplast numbers to C/CM2x^29^ (Figs. 2A, 4, 5, Tables 3, 4, Supplementary Tables S2, S3). Chloroplast enlargement in palisade mesophyll brought about by rootstock tetraploidy in 2x common clementine leaves was not associated with an increase in chloroplast numbers. This phenomenon appears to reduce photosynthetic capacity^38^. Overall, under optimal conditions and depending on the rootstock genotypes, the structural and/or ultrastructural modifications of the 2x common clementine leaves induced by rootstock tetraploidy could either compensate each other with no change in gas exchanges or induce a modification of gas exchanges. On the whole, the number and size of transitory starch grains and mitochondria in palisade and spongy mesophylls in 2x common clementine scion were either reduced or similar between 2x and 4x rootstock of all the scion rootstock combination (Figs. 4, 5, Tables 3, 4, Supplementary Tables S2, S3). The number of plastoglobuli increased in C/CM4x and C/CC4x compared to C/CM2x and C/CC2x in the chloroplasts of the palisade and spongy mesophylls. Only the C/CM4x plastoglobuli were larger than those of its 2x counterpart (Figs. 4, 5, Tables 3, 4, Supplementary Tables S2, S3). Thus, rootstock tetraploidy involved a potential increase in lipid (plastoquinone-9 (PQ-9), plastoquinol-9 (PQ-9H2) and a-tocopherol (a-T)) reserves that could not be deposited in the thylakoids of chloroplasts. Genotype and/or ploidy level of the rootstocks may influence the mineral content, structure and ultrastructure of 2x common clementine leaves.

Differences in photosynthetic properties and redox status of scion/rootstock combinations under nutrient stress could be related to their leaf and cell anatomy.

Complete starvation resulted in a significant decrease in N but similar or higher levels of P, K, Mg, Ca and Na than controls in all scion/rootstock combinations (Table 1). A concentration effect induced by the transfer of N to other tree areas could explain the increase in P, K and Mg contents in all scion/rootstock combinations^39^. Leaf chlorosis occurs when plants do not have the nutrients needed for chlorophyll synthesis, which in turn affects the photosynthetic efficiency^40^. However, the decrease in N, which was an important factor in chlorosis, was similar in all scion/rootstock combinations (Table 1). C/CM4x showed less chlorosis as indicated by its light green colour compared with the yellow colour of other scion/rootstock combinations (Fig. 1). Less pronounced chlorosis in C/CM4x is correlated with a slight decrease in chlorophyll content than in other the scion/rootstock combinations (Figs. 1, 2D).

These results suggest that, depending on the rootstock genotype, the tetraploidy may provide a better integrity of chlorophyll content probably related to a better protection against ROS in 2x common clementine scion under nutrient deficiency^41–43^. The improvements in the redox status of scions by tetraploid rootstocks is probably linked to an improvement in photosynthesis.

Chlorosis was associated with structural and ultrastructural foliar changes and a disruption of photosynthetic properties in all scion/rootstock combinations^6,44^ (Figs. 1, 2, 3, 4, 5, Tables 2, 3, 4, Supplementary Tables S1–S3). At structural level, stomata and ostioles of all scion/rootstock combinations showed a narrowing which was associated with an increase in density (except in C/CC2x) response to nutrient deficiency (Fig. 3, Table 2, Supplementary Table S1). The rootstock apparently has a different effect on the structural components of 2x common clementine leaves depending on genotype and/or ploidy level. *P*_net_ and *g*_s_ decreased concomitantly in all scion/rootstock combinations under nutrient deficiency (Fig. 2A,B). These results suggest a critical role of stomatal structure in the process of CO_2_ availability^45^. However, the smaller decrease in *P*_net_, *g*_s_ and *F*_v_/*F*_m_ in C/CM4x than in other scion/rootstock combinations (Fig. 2A–C) implies that other factors than stomata are needed to sustain photosynthesis^46–48^.

At ultrastructural level, nutrient deficiency resulted in slight cell size enlargement in palisade mesophyll, thylakoid with grana degradation and a decrease in chloroplast size in leaf mesophylls of all scion/rootstock combinations (Figs. 4, 5, Tables 3, 4, Supplementary Tables S2, S3). Similar results were recorded in other plants under conditions of high light stress, infection, dark-induced senescence or total nutrient deficiency^6,29,49,50^. Chloroplast degradation may be due to the significant decrease in N in the mature leaves of each scion/rootstock combination following the extensive remobilization of N present in the cell to younger leaves or storage areas during nutrient deficiency^51,52^ (Figs. 4, 5, Tables 1, 3, 4, Supplementary Tables S2, S3).

**Figure 6:**
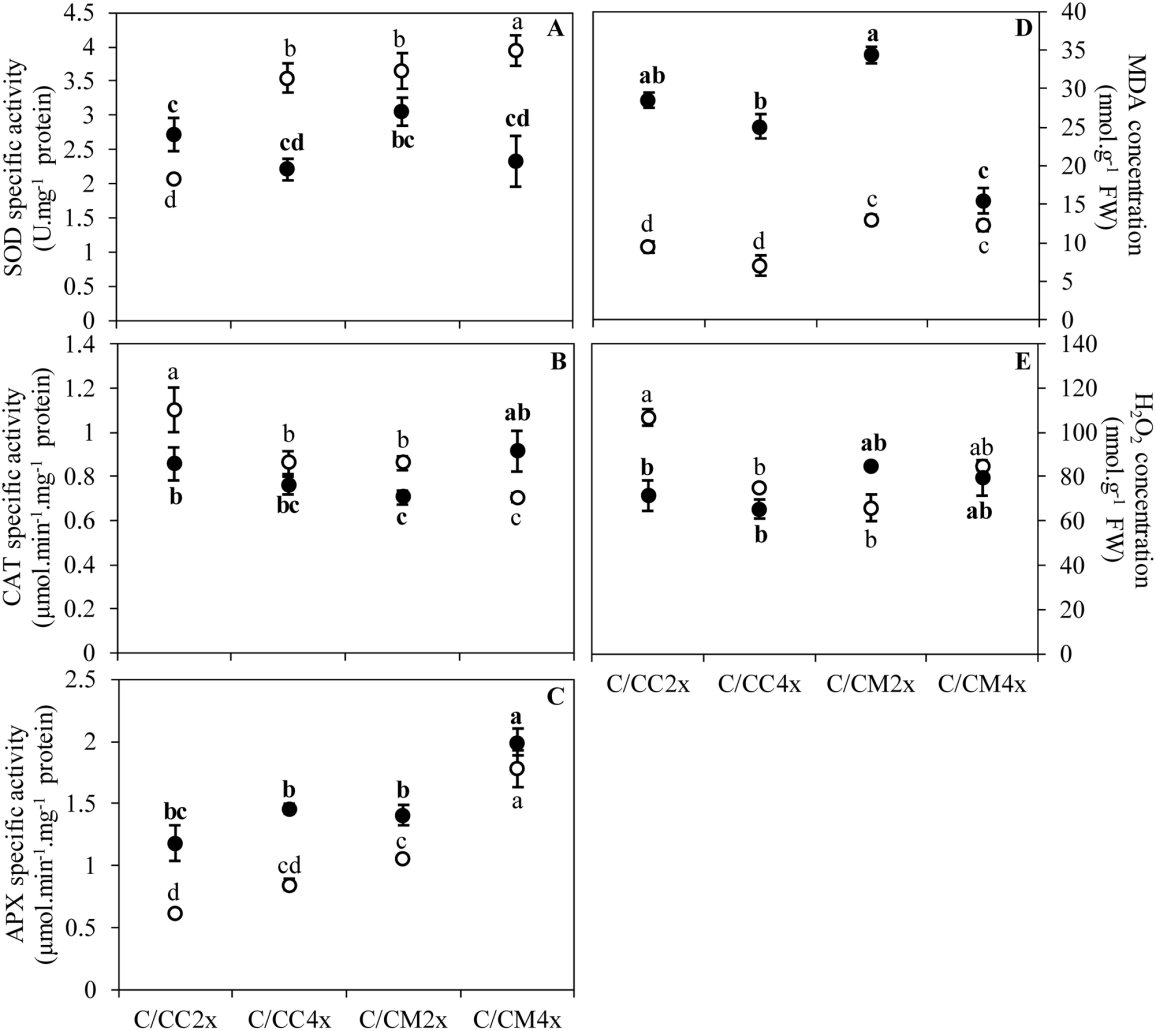
Effect of nutrient deficiency on antioxidant enzyme activities and contents in oxidative compounds in leaves of common clementine scion grafted onto different rootstocks. (**A**) Superoxide dismutase (SOD), (**B**) catalase (CAT) and (**C**) ascorbate peroxidase (APX) specific activities and (**D**) malondialdehyde (MDA) and (**E**) H_2_O_2_ contents in leaves of common clementine scion grafted onto diploid (C/CC2x) and doubled diploid (C/CC4x) Carrizo citrange and diploid (C/CM2x) and doubled diploid (C/CM4x) Citrumelo 4475 rootstocks grown in nutrient reference solution (100%) (white circles) and without nutrient solution (0%) (black circles) for 210 days. Values are mean (± standard error) of 3 independent measurements from 3 samples for each scion/rootstock combination, i.e. one per tree, obtained by pooling 8 fully-expanded leaves (*n* = 3). Ploidy and treatment effects were analyzed using ANOVA and Fisher LSD tests (*p* < 0.05). Distinct letters indicate significant differences between the four scion/rootstocks combinations and treatments.

The overproduction of reactive oxygen species (ROS) leads to alterations in the integrity of the membrane structures of cells, chloroplasts or thylakoids by a process called lipid peroxidation, one of the indicators of which is malondialdehyde (MDA). The lower MDA content in C/CM4x compared to other scion/rootstock combinations was consistent with the reduced damage to ultrastructure (Figs. 4, 5, 6D, Tables 3, 4, Supplementary Tables S2, S3). Thylakoid size and structure is dependent on the formation of PSII-LHCII supercomplexes^53^. In C/CM4x, the lower decrease in *F*_v_/*F*_m_ suggests that N decrease leads a more limited degradation of the PSII-LHCII supercomplexes reducing the disruption of electron transport for the photosynthetic reaction and thus the production of ROS in comparison to the other scion/rootstock combination^9,54,55^ (Figs. 2C, 6D)*.* Maintaining the redox balance in C/CM4x decelerates the degradation of N pool of the chlorophyll and thereby thylakoids that result in a more efficient photosynthetic capacity (*P*_net_ and *F*_v_/*F*_m_) (Figs. 2A,C,D, 4, 5, Tables 3, 4, Supplementary Table S2, S3). In addition, a lower allocation of N for Rubisco in support of membrane formation would explain the maintenance of chloroplast size and less advanced thylacoid degradation in C/CM4x. This phenomenon is consistent with the higher chlorophyll content in C/CM4x which would explain its more efficient regulation of photosynthesis^30^.

Plastoglobuli are bound to thylakoids via their membranes. Studies have shown that plastoglobuli are involved in the formation and degradation of thylakoids during plant growth, development and senescence but also when plants are exposed to drought, high-light, N starvation or fungal infection^50,56–58^. The reduced damage of the thylakoid membrane in C/CM4x would explain the smallest change or maintenance of the plastoglobuli numbers associated to a small increase in their size under N decrease due to the accumulation of catabolites formed by thylakoid degradation in their hydrophobic core compared to other scion/rootstock combinations^59–62^ (Figs. 4, 5, Tables 1, 3, 4, Supplementary Table S2, S3).

Tetraploidy of the Citrumelo 4475 rootstock improves the tolerance of 2x common clementine scions by maintaining the redox status and delaying ultrastructural changes and damage with a consequent improvement in photosynthetic capacity. However, as suggested by the similar impact of nutrient deficiency on C/CC2x and C/CC4x, the rootstock tetraploidy depending on their genotypes does not automatically result in better tolerance of the photosynthetic properties of the scion.

Better tolerance to nutrient stress in 2x common clementine scion grafted onto the 4x citrumelo 4475 rootstock may be explained by a better antioxidant system.

Nutrient deprivation led to a modification of oxidative metabolism^39,43,63^. Tolerance differences between scion/rootstock combinations may be related to differences in ROS production and/or antioxidant system efficiency. The enzymatic antioxidant system response differs according to plant species and the deficient minerals^63–67^. Enzymatic antioxidant systems in 2x common clementine leaves differed depending on the genotypes and/or the ploidy level of the rootstock (Fig. 6).

However, it was greater in C/CM4x than in the other scion/rootstock combinations.

Despite the decline in SOD activity and the increased or similar values in APX activity in all scion/rootstock combinations (Fig. 6A,C), CAT activity increased only in C/CM4x under nutrient deficiency (Fig. 6B). Concurrent CAT and APX activity is important for the elimination of H_2_O_2_^68–70^. In C/CM2x, C/CC2x and C/CC4x, the low H_2_O_2_ and high MDA contents were due to increased OH^·^ formation (Fig. 6D,E). This OH^·^ is either directly formed by the addition of electrons to the O_2_^·−^ not transformed into H_2_O_2_ by SOD and/or by the transformation of H_2_O_2_ via Fenton or Haber–Weiss reactions when the APX activity is insufficient to compensate for the low CAT activity. Conversely, in C/CM4x, the synergistic activity of CAT and APX maintains the MDA content in C/CM4x (Fig. 6B–D). As in their ungrafted counterparts, the increased enzymatic activity in response to nutrient deficiency may explain the reduced ultrastructural damage and decrease in photosynthetic activity in C/CM4x in comparison with the other scion/rootstock combinations. According to some study, the rootstock influences the scion performance by modifying their sources-sink relation^16,71,72^. A modification of gene expression in the roots of tetraploid Lime Rangpur (*Citrus limonia*, Osbeck) would regulate the source-sink relation between the rootstock and the scion improving the stress adaptation^25^.

Tetraploidy of the citrumelo 4475 would improve the performance of the enzyme-based antioxidant system and thus the photosynthetic activity in the 2x common clementine scion, likely to change the regulation of the sources sink relation.

In conclusion, rootstock ploidy level had no effect on the structure of the 2x common clementine scion leaves (except stomata density in Carrizo citrange) whereas it induced modifications in the ultrastructural components. The impact of prolonged nutrient deficiency on the structure, ultrastructure, physiology and biochemistry of the 2x common clementine scion differed according to the variety and ploidy level of the rootstock. Among the four scion/rootstock combinations, 2x common clementine grafted with 4x citrumelo 4475 rootstock (C/CM4x) was the most tolerant to nutrient deficiency as indicated by the limited changes in leaf cell structures and photosynthetic activity. The improved tolerance of 2x common clementine grafted with 4x citrumelo 4475 rootstock may be related to a better antioxidant system. Tolerance to nutrient deficiency can therefore be improved by rootstock polyploidy but remains dependent on the rootstock genotype. The next step of this study will be to test the impact of rootstock ploidy level on the quality and yield of clementine fruit under nutrient deficiency.

## Materials and methods

### Plant material and experimental design

The experiment was carried out on the AREFLEC experimental station located in San Giuliano, Corsica (41° 47′ 27′′ N and 09° 23′ 40′′ E). 2x common clementine (*Citrus clementina* Hort. ex Tan; SRA 92) scion grafted onto one year seedlings of Carrizo citrange (*Citrus sinensis* L. Osb*.* × *Poncirus trifoliata* L. Raf.) and Citrumelo 4475 (*Citrus paradisi* L. Macf. × *Poncirus trifoliata* L. Raf.) 2x (C/CC2x and C/CM2x, respectively) and their 4 × counterparts (C/CC4x and C/CM4x, respectively) were used as source materials. All, the citrus seeds have been provided by the INRAE-CIRAD of San Giuliano in Corsica (France) from the collection of the “CRB Citrus” biological resource center with the authorisation of Olivier Pailly, director of the INRAE Experimental Unit CITRUS of San Giuliano^73^. The INRAE-CIRAD center is recognized by the French Ministry of Agriculture, Food and Forestry. All the plant material mentioned comply with relevant institutional, national, and international guidelines and legislation. Subsequently, we cultivated the rootstocks and grafted them with the common clementine. The ploidy status of six seedlings for each combination was first checked by flow 10 cytometry (Partec I, Germany)^74^. Clonal propagation by nucellar embryogenesis was checked by genotyping using SSR markers^75^.

The four selected scion/rootstock combinations were then grown under identical conditions in vermiculite with fertigation and water (1 L/h) for 3 years in a tunnel greenhouse. The stock solution used for irrigation included: 20-5-10 NPK + 2MgO fertilizer + trace elements according to the recommendations of the French department of agriculture. Seedlings were divided into two blocks: one with reference fertigation (control plants) and the other with irrigation water (without nutrient inputs). A total of three plants of each scion/rootstock combination were randomized by fertigation level (*n* = 3). The fertigation solutions were prepared and applied with a metering pump. Before starting the experiment, the vermiculite was washed for 48 h in order to eliminate any nutritional reserves in the pot.

According to a previous experiment^29^, leaf samples were collected and physiological measurements made from May to December 2018 at two different times (days): 0 (D0: control plant) and 210 (D210) days after the start of nutritional deprivation. Measurements were made and samples taken from homogeneous plants comprising four branches with fully-expanded leaves developed under stress and control conditions.

Mineral content was measured on a pool of eight fully expanded leaves for the three plants per combination and fertigation level (*n* = 3) between 10:00 and 11:00 am. Fresh leaves were placed in a forced air oven at 65 ± 10 °C overnight and then transferred into a desiccator for cooling. The dehydrated leaves were then sent to the CIRAD “Analyses des eaux, sols et végétaux service unit” at Montpellier (France) for analysis of macro- and micro-nutrients.

Leaf P, K, Ca, Mg and Na contents were measured using an Agilent 720 simultaneous ICP-OES after double calcination with silica removal by adding hydrofluoric acid.

The leaf total N content was evaluated after combustion using a Leco TruMac N determinator.

### Scanning electron microscopy (SEM)

Scanning electron microscopy measurements were carried out on three leaf pieces per scion/rootstock combination and fertigation level (typically 1 cm^2^) (*n* = 3) cut with a razor blade from mid-laminar areas at between 10:00 and 11:00 am. Leaves were then immediately fixed in cold (4 °C) 2.5% (v/v) glutaraldehyde in 0.1 M sodium cacodylate buffer at pH 7.2, rinsed in a 0.1 M cacodylate buffer at pH 7.2, dehydrated through a graded ethanol series (30%, 50%, 75%, 90% and 100%) and dried under CO_2_ in an Emitech K850 critical point dryer (Quorum Technologies Ltd, Ashford, U.K.)^29^. Specimens were mounted on aluminum stubs with carbon double-sided adhesive disks, coated with gold/palladium in a SC7640 sputter coater (Quorum Technologies Ltd, Newhaven, U.K.) and examined under a S-3400N scanning electron microscope (Hitachi High-Technologies Corporation, Tokyo, Japan) at an accelerating voltage of 5 kV.

### Transmission electron microscopy (TEM)

Transmission electron microscopy measurements were carried out on five leaf pieces per scion/rootstock combination and fertigation level (typically 1 mm^2^) (*n* = 5) cut with a razor blade from mid-laminar areas at between 10:00 and 11:00 am. Leaves were immediately fixed in cold (4 °C) 2.5% glutaraldehyde in 0.1 M sodium cacodylate buffer at pH 7.2, rinsed in a 0.1 M cacodylate buffer at pH 7.2, post-fixed in cold (4 °C) 1% osmium tetroxide in the same buffer for 1 h, dehydrated through a graded ethanol series (70% and 100%) and propylene oxide, embedded in Spurr, and polymerized at 60 °C for 24 h^29^. Ultra-thin sections (60–90 nm) were cut using a Power tome PC ultramicrotome (RMC Boeckeler, Tuscon, U.S.A.). Sections were placed on 200- and 300-mesh copper grids and stained with UranyLess (Delta Micoscopies, France) and lead citrate. They were then examined using a Hitachi H-7650 (Hitachi High-Technologies Corporation, Tokyo, Japan) at an accelerating voltage of 80 kV.

### Measurements of gas exchange, chlorophyll content and chlorophyll a fluorescence

All measurements were made on three fully developed leaves for each of the three plants per combination and fertigation level (*n* = 9). A portable photosynthesis system (LI600) was used to measure the leaf net photosynthetic rate (*P*_net_), stomatal conductance (*g*_s_) and transpiration rate (E) at between 7:00 and 11:00 am. The carbon dioxide concentration (CO_2_), airflow rate, light intensity and temperature of the leaf chamber were maintained at 380 μmol mol^−1^, 500 μmol s^−1^, 1400 μmol m^−2^ s^−1^ and 25 °C, respectively.

The chlorophyll content was measured non-invasively with a Dualex Scientific + meter (FORCE-A, Paris). Values were obtained in Dualex units, convertibles to g cm^−2^.

Chlorophyll *a* fluorescence parameters were measured using an OS1p (Hansatech, Instruments Ltd) at between 9:00 and 11:00 am. Leaves were dark-acclimated for 30 min using special leaf clips. Chlorophyll a fluorescence was recorded after illumination with red actinic light (650 nm, 3000 μmol photon m^−2^ s^−1^) for 1 s and this was used to calculate the maximum fluorescence [*F*_v_/*F*_m_ = (*F*_m_ − *F*_o_)/F_m_]^76^. Leaves were exposed to an actinic light to evaluate the current fluorescence yield (*F*_s_) and the actual light-adapted fluorescence (*F*_m_′). Formulas were applied to this data in order to determine the effective quantum yield of PSII Y(II) = (*F*_m_’ − *F*_*s*_)/*F*_m_’], the Y(NO) = *F*_s_/*F*_m_], the non-photochemical quenching coefficient [Y(NPQ) = (*F*_s_/*F*_m_’)-Y(NO)]^77,78^, and the electron transport rate through PSII [ETR(II) = Y(II) × PAR × 0.5 × 0.84]^79^. The ETR/*P*_net_ ratio was calculated to estimate the use of electrons in other processes unrelated to the photosynthetic CO_2_ assimilation rate.

### Determination of oxidative stress and antioxidant levels

Biochemical analyses were performed on three samples for each scion/rootstock combination, i.e. one per tree, obtained by pooling eight fully-expanded leaves (*n* = 3) collected between 10:00 and 11:00 am and immediately immersed in liquid nitrogen and stored at − 80 °C. Immediately prior to biochemical analysis, each leaf was ground to a fine powder in liquid nitrogen.

Malondialdehyde (MDA) and antioxidant enzyme activities (SOD, CAT, and APX) were assayed as defined by Santini et al.^80^.

Hydrogen peroxide (H_2_O_2_) was assayed using the PeroxiDetect kit (Sigma-Aldrich). This technique is based on the oxidation of ferrous (Fe^2+^) to ferric ions (Fe^3+^) by hydroperoxides which react with xylenol orange (“3,3′-bis[N,N-bis(carboxymethyl)aminomethyl] o-cresolsulfonephthalein, sodium salt”) to form a blue complex visible at 560 nm.

Proline content was measured as described by Oustric et al.^81^.

A V-630 spectrophotometer was used for all measurements (Jasco Inc., Tokyo, Japan).

### Statistical analyses

All statistical measurements were performed with R statistical software (v.2.12.1) (http://www.R-project.org) and the Rcmdr package. The qualitative factors studied were sampling date (D0 and D210 after nutrient deficiency), the clementine scion grafted onto rootstocks subjected to nutrient stress (C/CC and C/CM) and the ploidy level of nutrient stressed rootstocks (C/CC2x, C/CC4x, C/CM2x and C/CM4x). The influence of these three factors was analyzed using a two-way ANOVA followed by LSD test at *p* < 0.05 for photosynthetic parameters (Fig. 2), microscopic parameters (Supplementary Tables S1–S3) and antioxidant enzyme activities and contents in oxidative compounds (Fig. 6).

To investigate microscopic profile of the clementine scion overs, the sampling date, the nutrient stressed rootstock genotypes and their ploidy level, the microscopic data were represented as a heatmap to facilitate visualization (Tables 2, 3, 4). Both heatmaps and hierarchical cluster analysis were generated by Heatmap.2 function of the gplot package 3.0.1 for Rstudio (v.1.3.1093) (https://rstudio.com).

## Supplementary Information

Supplementary Information.
