# Supplementary material for: Tetraploid Citrumelo 4475 rootstocks improve diploid common clementine tolerance to long-term nutrient deficiency
Source: Sci Rep. 2021 Apr 26;11:8902. doi: 10.1038/s41598-021-88383-5 (PMC8076223; doi:10.1038/s41598-021-88383-5)
Supplement: Supplementary file 1 — Supplementary Information. [file 41598_2021_88383_MOESM1_ESM.docx]

**Supplementary data**

**Supplementary Table S1**

|  |  |  |  |  |  |  |  |  |
| --- | --- | --- | --- | --- | --- | --- | --- | --- |
| **Characters** | **C/CC2x** | | **C/CC4x** | | **C/CM2x** | | **C/CM4x** | |
|  | **100%** | 0% | **100%** | 0% | **100%** | 0% | **100%** | 0% |
| Position of stomata | Hypostomatic | | | | | | | |
| Stomatal type | Anomocytic | | | | | | | |
| Stomatal density | **24.46 ± 0.65^ab^** | 24.78 ± 0.53^ab^ | **18.26 ± 0.56^c^** | 23.60 ± 0.49^ab^ | **22.06 ± 0.15^b^** | 26.60 ± 0.47^a^ | **21.80 ± 0.45^b^** | 25.53 ± 0.46^a^ |
| stomata (μm^2^) | **351.93 ± 47.82^b^** | 316.09 ± 40.68^cd^ | **349.59 ± 47.98^b^** | 309.96 ± 67.03^d^ | **374.61 ± 42.65^a^** | 332.45 ± 53.80^c^ | **371.48 ± 46.71^a^** | 304.39 ± 52.79^d^ |
| ostiole (μm^2^) | **26.88 ± 8.96^ab^** | 21.50 ± 7.33^de^ | **29.86 ± 8.87^a^** | 24.60 ± 8.26^cd^ | **28.81 ± 7.38^ab^** | 25.56 ± 8.87^bc^ | **27.91 ± 6.72^ab^** | 18.64 ± 6.86^e^ |
| Trichome type | Absent | | | | | | | |
|  |  |  |  |  |  |  |  |  |

Anatomical characters of leaves epidermis of common clementine scion grafted onto diploid (C/CC2x) and doubled diploid (C/CC4x) Carrizo citrange and [diploid](https://www.sciencedirect.com/topics/biochemistry-genetics-and-molecular-biology/diploidy) (C/CM2x) and doubled diploid (C/CM4x) Citrumelo 4475 rootstocks grown in nutrient reference solution (100%) and without nutrient solution (0%) for 210 days. For [length](https://www.sciencedirect.com/topics/biochemistry-genetics-and-molecular-biology/length) and [width](https://www.sciencedirect.com/topics/agricultural-and-biological-sciences/width) of [stomata](https://www.sciencedirect.com/topics/biochemistry-genetics-and-molecular-biology/stoma) and ostioles, values are means (± standard error) of 30 independent [measurements](https://www.sciencedirect.com/topics/agricultural-and-biological-sciences/measurement) on three different [leaves](https://www.sciencedirect.com/topics/agricultural-and-biological-sciences/leaves) (*n* = 90). For stomatal density, values are means of five independent measurements on three different leaves (*n* = 15) for stomatal [density](https://www.sciencedirect.com/topics/biochemistry-genetics-and-molecular-biology/density). Data were analyzed using ANOVA and Fisher LSD tests (*p* < 0.05). Different letters indicate significant differences between the four scion/rootstock combinations and treatments.

**Supplementary Table S2**

|  |  |  | |  | |  | |  | |  | |  | |  | |  | |  |
| --- | --- | --- | --- | --- | --- | --- | --- | --- | --- | --- | --- | --- | --- | --- | --- | --- | --- | --- |
| **Characters** |  | | **C/CC2x** | | | | **C/CC4x** | | | | **C/CM2x** | | | | **C/CM4x** | | | |
|  |  | | **Palisade mesophyll cells** | | | | | | | | | | | | | | | |
|  |  | | **100%** | | 0% | | **100%** | | 0% | | **100%** | | 0% | | **100%** | | 0% | |
| Cells | Length (μm) | | **27.41 ± 3.83^b^** | | 30.24 ± 4.03^ab^ | | **20.68 ± 3.49^c^** | | 32.80 ± 2.51^ab^ | | **25.40 ± 2.81^bc^** | | 32.47 ± 3.86^ab^ | | **30.83 ± 4.47^ab^** | | 34.33 ± 3.80^a^ | |
|  | Width (μm) | | **8.37 ± 1.24^ab^** | | 10.59 ± 0.97^a^ | | **7.31 ± 1.27^b^** | | 9.50 ± 1.56^ab^ | | **6.93 ± 1.28^c^** | | 6.60 ± 1.58^c^ | | **9.01 ± 1.04^ab^** | | 10.08 ± 1.48^a^ | |
|  | Thickness (μm) | | **0.40 ± 0.06^bc^** | | 0.39 ± 0.05^bc^ | | **0.30 ± 0.04^d^** | | 0.57 ± 0.02^a^ | | **0.35 ± 0.84^cd^** | | 0.34 ± 0.06^cd^ | | **0.43 ± 0.09^b^** | | 0.43 ± 0.02^b^ | |
| Chloroplasts | Number | | **7.40 ± 1.20^ab^** | | 4.40 ± 1.02^cd^ | | **5.60 ± 1.74^bc^** | | 3.00 ± 1.09^d^ | | **6.00 ± 1.02^bc^** | | 6.40 ± 2.07^b^ | | **6.60 ± 1.02^b^** | | 9.60 ± 1.74^a^ | |
|  | Length (μm) | | **5.66 ± 1.34^bc^** | | 4.20 ± 0.78^d^ | | **4.49 ± 1.04^d^** | | 4.05 ± 0.91^d^ | | **5.01 ± 1.35^cd^** | | 4.92 ± 0.76^cd^ | | **7.13 ± 2.05^a^** | | 6.70 ± 1.62^ab^ | |
|  | Width (μm) | | **2.66 ± 0.46^b^** | | 1.78 ± 0.34^c^ | | **2.65 ± 0.29^b^** | | 1.80 ± 0.54^c^ | | **2.99 ± 1.03^b^** | | 2.77 ± 0.39^b^ | | **3.60 ± 0.70^a^** | | 2.86 ± 0.38^b^ | |
| Starches | Number | | **11.80 ± 2.48^bc^** | | 7.40 ± 2.24^c^ | | **12.00 ± 2.65^bc^** | | 3.60 ± 1.02^d^ | | **15.60 ± 2.58^a^** | | 14.40 ± 5.73^ab^ | | **12.20 ± 4.44^bc^** | | 13.00 ± 2.90^b^ | |
|  | Length (μm) | | **1.03 ± 0.44^d^** | | 1.93 ± 0.69^bc^ | | **1.59 ± 0.69^cd^** | | 2.78 ± 1.26^a^ | | **2.32 ± 0.68^ab^** | | 2.62 ± 0.82^a^ | | **1.32 ± 0.32^d^** | | 1.98 ± 0.72^bc^ | |
|  | Width (μm) | | **0.72 ± 0.27^d^** | | 1.06 ± 0.41^bc^ | | **0.96 ± 0.43^cd^** | | 1.06 ± 0.53^bc^ | | **1.33 ± 0.42^ab^** | | 1.64 ± 0.37^a^ | | **0.92 ± 0.28^cd^** | | 1.20 ± 0.50^b^ | |
| Plastoglobuli | Number | | **31.60 ± 6.82^bc^** | | 12.40 ± 4.27^c^ | | **39.60 ± 7.20^b^** | | 4.80 ± 0.75^d^ | | **17.00 ± 3.16^c^** | | 38.60 ± 12.62^b^ | | **55.20 ± 10.79^a^** | | 39.60 ± 6.28^b^ | |
|  | Length (μm) | | **0.40 ± 0.09^c^** | | 0.55 ± 0.14^b^ | | **0.31 ± 0.06^d^** | | 0.89 ± 1.60^a^ | | **0.33 ± 0.08^d^** | | 0.63 ± 0.10^ab^ | | **0.46 ± 0.12^bc^** | | 0.59 ± 0.04^ab^ | |
|  | Width (μm) | | **0.31 ± 0.07^b^** | | 0.52 ± 0.05^ab^ | | **0.23 ± 0.06^c^** | | 0.59 ± 0.11^a^ | | **0.28 ± 0.06^b^** | | 0.44 ± 0.11^b^ | | **0.31 ± 0.09^b^** | | 0.37 ± 0.09^b^ | |
| Mitochondria | Number | | **19.00 ± 3.69^a^** | | 7.40 ± 2.65^bc^ | | **13.60 ± 1.35^ab^** | | 4.80 ± 0.98^c^ | | **18.00 ± 2.44^a^** | | 8.00 ± 1.58^bc^ | | **18.50 ± 2.95^a^** | | 15.40 ± 4.18^a^ | |
|  | Length (μm) | | **0.84 ± 0.19^b^** | | 0.76 ± 0.26^bc^ | | **0.75 ± 0.12^bc^** | | 0.74 ± 0.27^bc^ | | **0.93 ± 0.19^a^** | | 0.60 ± 0.13^c^ | | **0.89 ± 0.26^b^** | | 0.98 ± 0.24^a^ | |
|  | Width (μm) | | **0.66 ± 0.12^a^** | | 0.57 ± 0.13^b^ | | **0.57 ± 0.08^b^** | | 0.51 ± 0.15^bc^ | | **0.68 ± 0.14^a^** | | 0.46 ± 0.09^c^ | | **0.59 ± 0.16^b^** | | 0.62 ± 0.16^ab^ | |
| Grana | Number of grana per cell | | **30.70 ± 5.69^a^** | | Absent | | **20.47 ± 3.56^b^** | | Absent | | **24.23 ± 2.97^b^** | | Absent | | **30.87 ± 9.31^a^** | | Absent | |
|  | Length (μm) | | **0.33 ± 0.08^a^** | | Absent | | **0.36 ± 0.13^a^** | | Absent | | **0.36 ± 0.08^a^** | | Absent | | **0.39 ± 0.13^a^** | | Absent | |
|  | Width (μm) | | **0.35 ± 0.11^b^** | | Absent | | **0.50 ± 0.14^a^** | | Absent | | **0.39 ± 0.12^b^** | | Absent | | **0.42 ± 0.13^ab^** | | Absent | |
|  |  |  | |  | |  | |  | |  | |  | |  | |  | |  |

Ultrastructure characters of palisade [mesophyll](https://www.sciencedirect.com/topics/agricultural-and-biological-sciences/mesophyll) [cells](https://www.sciencedirect.com/topics/agricultural-and-biological-sciences/cells) of leaves of common clementine scion grafted onto diploid (C/CC2x) and doubled diploid (C/CC4x) Carrizo citrange and [diploid](https://www.sciencedirect.com/topics/biochemistry-genetics-and-molecular-biology/diploidy) (C/CM2x) and doubled diploid (C/CM4x) Citrumelo 4475 rootstocks grown in nutrient reference solution (100%) and without nutrient solution (0%) for 210 days. Values are means (± standard error) of independent [measurements](https://www.sciencedirect.com/topics/agricultural-and-biological-sciences/measurement) on five different cells section (*n* = 5) for [length](https://www.sciencedirect.com/topics/biochemistry-genetics-and-molecular-biology/length), [width](https://www.sciencedirect.com/topics/agricultural-and-biological-sciences/width) and [thickness](https://www.sciencedirect.com/topics/biochemistry-genetics-and-molecular-biology/thickness) of cells and for number of [chloroplasts](https://www.sciencedirect.com/topics/biochemistry-genetics-and-molecular-biology/chloroplast), [starches](https://www.sciencedirect.com/topics/biochemistry-genetics-and-molecular-biology/starch), plastoglobuli and [mitochondria](https://www.sciencedirect.com/topics/biochemistry-genetics-and-molecular-biology/mitochondrion) and of 30 independent measurements on different cells section (*n* = 30) for [length](https://www.sciencedirect.com/topics/agricultural-and-biological-sciences/length) and width of [chloroplasts](https://www.sciencedirect.com/topics/agricultural-and-biological-sciences/chloroplast), [starches](https://www.sciencedirect.com/topics/agricultural-and-biological-sciences/starch), plastoglobuli, [mitochondria](https://www.sciencedirect.com/topics/agricultural-and-biological-sciences/mitochondrion), [grana](https://www.sciencedirect.com/topics/agricultural-and-biological-sciences/grana), number of grana per cells section and number of [thylakoids](https://www.sciencedirect.com/topics/biochemistry-genetics-and-molecular-biology/thylakoid) per granum. Data were analyzed using ANOVA and Fisher LSD tests (*p* < 0.05). Different letters indicate significant differences between the four scion/rootstock combinations and treatments.

**Supplementary Table S3**

|  |  |  |  |  |  |  |  |  |  |  |
| --- | --- | --- | --- | --- | --- | --- | --- | --- | --- | --- |
| **Characters** |  |  | **C/CC2x** | | **C/CC4x** | | **C/CM2x** | | **C/CM4x** | |
|  |  |  | **Spongy mesophyll cells** | | | | | | | |
|  |  |  | **100%** | 0% | **100%** | 0% | **100%** | 0% | **100%** | 0% |
| Cells | Length (μm) | | **24.43 ± 4.50^ab^** | 26.33 ± 2.05^ab^ | **18.99 ± 2.02^b^** | 33.43 ± 6.86^a^ | **18.21 ± 1.47^b^** | 17.65 ± 3.28^b^ | **23.00 ± 4.32^ab^** | 25.33 ± 4.60^ab^ |
|  | Width (μm) | | **20.80 ± 3.80^ab^** | 16.94 ± 4.28^ab^ | **15.47 ± 1.01^b^** | 24.13 ± 4.81^a^ | **14.03 ± 1.73^b^** | 13.34 ± 1.48^b^ | **17.75 ± 3.75^ab^** | 16.77 ± 3.81^ab^ |
|  | Thickness (μm) | | **0.83 ± 0.20^ab^** | 0.82 ± 0.13^ab^ | **0.49 ± 0.14^c^** | 0.74 ± 0.22^b^ | **0.66 ± 0.13^bc^** | 0.43 ± 0.11^c^ | **0.83 ± 0.21^ab^** | 1.01 ± 0.12^a^ |
| Chloroplasts | Number | | **5.80 ± 0.75^a^** | 3.60 ± 1.02^ac^ | **3.80 ± 0.40^ac^** | 2.20 ± 0.98^c^ | **4.70 ± 0.82^ab^** | 3.20 ± 1.92^bc^ | **5.00 ± 0.70^ab^** | 5.40 ± 1.02^ab^ |
|  | Length (μm) | | **6.75 ± 1.66^ab^** | 4.09 ± 1.03^de^ | **5.63 ± 1.00^bc^** | 3.71 ± 0.80^e^ | **6.37 ± 1.16^ab^** | 4.84 ± 0.96^cd^ | **6.95 ± 1.42^a^** | 6.20 ± 1.12^ab^ |
|  | Width (μm) | | **3.50 ± 0.64^a^** | 1.82 ± 0.64^b^ | **3.36 ± 0.74^a^** | 1.66 ± 0.47^b^ | **3.84 ± 0.83^a^** | 3.10 ± 0.49^ab^ | **3.21 ± 0.85^a^** | 2.79 ± 0.80^ab^ |
| Starches | Number | | **12.40 ± 2.65^a^** | 5.60 ± 0.49^bc^ | **9.20 ± 0.75^ab^** | 2.40 ± 1.85^c^ | **12.00 ± 1.67^a^** | 12.00 ± 3.39^a^ | **9.60 ± 1.85^ab^** | 11.80 ± 3.31^a^ |
|  | Length (μm) | | **3.23 ± 1.03^a^** | 2.29 ± 1.22^bc^ | **2.85 ± 0.47^ab^** | 1.65 ± 0.70^c^ | **3.41 ± 1.12^a^** | 2.63 ± 0.91^ab^ | **3.36 ± 1.19^a^** | 2.67 ± 1.04^ab^ |
|  | Width (μm) | | **1.94 ± 0.63^ab^** | 1.08 ± 0.81^cd^ | **2.04 ± 0.51^ab^** | 0.85 ± 0.52^d^ | **2.18 ± 0.68^a^** | 1.66 ± 0.47^b^ | **2.19 ± 0.99^a^** | 1.56 ± 0.64^bc^ |
| Plastoglobuli | Number | | **5.00 ± 1.58^d^** | 6.60 ± 1.62^cd^ | **7.67 ± 0.47^c^** | 10.20 ± 3.43^b^ | **9.20 ± 3.65^bc^** | 11.40 ± 3.78^ab^ | **12.60 ± 3.67^a^** | 12.00 ± 3.10^ab^ |
|  | Length (μm) | | **0.33 ± 0.07^d^** | 0.52 ± 0.08^b^ | **0.28 ± 0.08^d^** | 0.49 ± 0.12^c^ | **0.30 ± 0.04^d^** | 0.77 ± 0.19^a^ | **0.51 ± 0.11^b^** | 0.62 ± 0.80^ab^ |
|  | Width (μm) | | **0.28 ± 0.07^d^** | 0.38 ± 0.10^bc^ | **0.23 ± 0.08^de^** | 0.40 ± 0.08^bc^ | **0.18 ± 0.07^e^** | 0.58 ± 0.16^a^ | **0.39 ± 0.10^bc^** | 0.46 ± 0.12^b^ |
| Mitochondria | Number | | **12.80 ± 6.11^a^** | 7.00 ± 1.41^b^ | **6.20 ± 3.65^b^** | 4.40 ± 0.49^c^ | **10.00 ± 2.61^ab^** | 5.40 ± 3.85^c^ | **6.00 ± 1.41^b^** | 11.00 ± 2.76^ab^ |
|  | Length (μm) | | **0.73 ± 0.23^b^** | 0.71 ± 0.17^b^ | **0.71 ± 0.22^b^** | 0.73 ± 0.30^b^ | **0.65 ± 0.21^b^** | 0.67 ± 0.24^b^ | **0.97 ± 0.29^a^** | 0.78 ± 0.20^b^ |
|  | Width (μm) | | **0.57 ± 0.15^a^** | 0.48 ± 0.10^b^ | **0.52 ± 0.12^ab^** | 0.54 ± 0.15^ab^ | **0.56 ± 0.09^a^** | 0.43 ± 0.13^b^ | **0.59 ± 0.13^a^** | 0.54 ± 0.14^ab^ |
| Grana | Number of grana per cell | | **13.10 ± 1.51^b^** | Absent | **18.27 ± 3.55^a^** | Absent | **14.97 ± 2.87^b^** | Absent | **18.84 ± 2.66^a^** | Absent |
|  | Length (μm) | | **0.50 ± 0.17^a^** | Absent | **0.41 ± 0.07^b^** | Absent | **0.46 ± 0.06^ab^** | Absent | **0.52 ± 0.10^a^** | Absent |
|  | Width (μm) | | **0.69 ± 0.10^a^** | Absent | **0.56 ± 0.14^b^** | Absent | **0.40 ± 0.06^d^** | Absent | **0.48 ± 0.16^c^** | Absent |
|  |  |  |  |  |  |  |  |  |  |  |

Ultrastructure characters of spongy [mesophyll](https://www.sciencedirect.com/topics/agricultural-and-biological-sciences/mesophyll) [cells](https://www.sciencedirect.com/topics/agricultural-and-biological-sciences/cells) of leaves of common clementine scion grafted onto diploid (C/CC2x) and doubled diploid (C/CC4x) Carrizo citrange and [diploid](https://www.sciencedirect.com/topics/biochemistry-genetics-and-molecular-biology/diploidy) (C/CM2x) and doubled diploid (C/CM4x) Citrumelo 4475 rootstocks grown in nutrient reference solution (100%) and without nutrient solution (0%) for 210 days. Values are means (± standard error) of independent [measurements](https://www.sciencedirect.com/topics/agricultural-and-biological-sciences/measurement) on five different cells section (*n* = 5) for [length](https://www.sciencedirect.com/topics/biochemistry-genetics-and-molecular-biology/length), [width](https://www.sciencedirect.com/topics/agricultural-and-biological-sciences/width) and [thickness](https://www.sciencedirect.com/topics/biochemistry-genetics-and-molecular-biology/thickness) of cells and for number of [chloroplasts](https://www.sciencedirect.com/topics/biochemistry-genetics-and-molecular-biology/chloroplast), [starches](https://www.sciencedirect.com/topics/biochemistry-genetics-and-molecular-biology/starch), plastoglobuli and [mitochondria](https://www.sciencedirect.com/topics/biochemistry-genetics-and-molecular-biology/mitochondrion) and of 30 independent measurements on different cells section (*n* = 30) for [length](https://www.sciencedirect.com/topics/agricultural-and-biological-sciences/length) and width of [chloroplasts](https://www.sciencedirect.com/topics/agricultural-and-biological-sciences/chloroplast), [starches](https://www.sciencedirect.com/topics/agricultural-and-biological-sciences/starch), plastoglobuli, [mitochondria](https://www.sciencedirect.com/topics/agricultural-and-biological-sciences/mitochondrion), [grana](https://www.sciencedirect.com/topics/agricultural-and-biological-sciences/grana), number of grana per cells section and number of [thylakoids](https://www.sciencedirect.com/topics/biochemistry-genetics-and-molecular-biology/thylakoid) per granum. Data were analyzed using ANOVA and Fisher LSD tests (*p* < 0.05). Different letters indicate significant differences between the four scion/rootstock combinations and treatments.
